# Ethnic differences in names in China: A comparison between Chinese Mongolian and Han Chinese cultures in Inner Mongolia

**DOI:** 10.12688/f1000research.76837.1

**Published:** 2022-01-18

**Authors:** Yuji Ogihara

**Affiliations:** 1 Tokyo University of Science, Tokyo, Japan

**Keywords:** ethnic difference, name, China, Mongol, individualism, culture, uniqueness, cultural change

## Abstract

I propose two suggestions on Stojcic et al.’s (2020) Study 3, which examined ethnic differences in individualism between Chinese Mongolian and Han Chinese cultures in China. The authors analyzed the names of all residents in the Inner Mongolia Autonomous Region of China and found that the percentages of common names among Chinese Mongolians were smaller than those among Han Chinese. The authors concluded that Chinese Mongolians are more independent than Han Chinese. However, two questions remain unanswered. First, although the authors analyzed the names of people in all age groups together and did not analyze the names by birth year, how was the effect of time controlled? Second, although the authors treated name indices, which have been used as group-level indicators in previous research, as individual-level indicators, how did the authors confirm whether name indices can be used as individual-level indicators? Addressing these two questions would contribute to a better understanding of ethnic differences in individualism in China.

Stojcic et al. (2020) examined ethnic differences in individualism between Chinese Mongolian and Han Chinese in China. In Studies 1 and 2, the authors conducted surveys and investigated ethnic differences in independence and interdependence. In Study 3, they analyzed the names of all residents in the Inner Mongolia Autonomous Region of China and found that the percentages of common names among the Chinese Mongolians were smaller than those among Han Chinese. Based on a previous study showing that seeking unique names is an indicator of individualism ( Varnum & Kitayama, 2011), they concluded that Mongolians are more independent than Han Chinese.

I have conducted research on names. For example, I demonstrated that unique names increased in Japan ( Ogihara, 2021a, 2022a; Ogihara et al., 2015), described the characteristics and patterns of uncommon names in Japan ( Ogihara, 2015, 2021b), and explained difficulties in reading recent Japanese names ( Ogihara, 2021c, 2022c) and availability of name data in Japan ( Ogihara, 2020a, 2022b). I also revealed related cultural changes in Japan such as an increase in individualism (e.g., Ogihara, 2017b, 2018b; for reviews see, Ogihara, 2017a, 2018a) and a decrease in self-esteem (e.g., Ogihara, 2016; Ogihara et al., 2016; for a review see, Ogihara, 2017a). Further, there is a space constraint here. Thus, in this article, I focus on Study 3 conducted by Stojcic et al. (2020) and pose two questions that I suggest the authors address.

## 1. How was the effect of time controlled?

 The authors calculated the percentages of the top 1, 10, and 20 most common names by ethnic group and found that those among Chinese Mongolians were smaller than those among Han Chinese. ^1^ They concluded that Chinese Mongolians are more independent than Han Chinese.

However, the names of people in all age groups were analyzed together and were not analyzed by birth year. Although the specific age span of the population was unclear, they wrote that “we included data for Chinese Han and Mongolian living in the same area, i.e., the region of Inner Mongolia, over the span of some 60 years” (p. 7). A name of a person aged 60 years indicates naming behavior approximately 60 years ago. Thus, naming behaviors for more than 60 years were included in the data. This approach is problematic for comparison between ethnicities for at least four reasons.

First, the authors’ analyses did not exclude the possibility that the differences stemmed from the naming behaviors at different time points rather than ethnic differences at a specific time point. In other words, the authors’ analyses may not have compared the behaviors at the same time point. Considering that a prior study insisted that unique names increased over time in China ( Cai et al., 2018; but also see Ogihara, 2020b), this confounding could have affected the results. For example, younger people who have unique names may have been included in the analyses more in Chinese Mongolians than in Han Chinese. This may have caused the result that the percentages of the common names among Chinese Mongolians were smaller than those among Han Chinese. Moreover, distribution of age may have differed between the two ethnic groups.

Second, it is unclear what the indicators mean. The authors calculated the percentages of the top 1, 10, and 20 most common names over a period of more than 60 years rather than the common names by year. However, common names can drastically change over time. It is possible that common names 60 years ago are no longer common in the present. The meaning of common names in a given year is clear, but the meaning of names common over a period of more than 60 years is ambiguous.

Third, the analyses contradicted the authors’ claim that past sustenance styles (Mongolian: herding, Han Chinese: farming) affect “present” psychological tendencies (Mongolian: independence/individualism, Han Chinese: interdependence/collectivism). The authors emphasized present psychological tendencies, but they examined past psychological tendencies from more than 60 years ago.

Fourth, Study 3 examined different constructs than those in Studies 1 and 2. The authors stated that “in order to furthermore increase the ecological validity of the current research, in Study 3 we tested our hypothesis in real life setting by investigating the baby naming practices between the Chinese Han and Mongolian” (p. 6). However, in Study 3 the authors analyzed indicators for over 60 years, which was inconsistent with Studies 1 and 2, in which the authors measured psychological tendencies in recent years. ^2^

All of the previous studies that the authors cited in the article controlled this effect by analyzing indicators by year ( Ogihara et al., 2015; Twenge et al., 2010) or at the specific year (2007; Varnum & Kitayama, 2011). Thus, analyzing data in recent years (preferably the year when the data were collected in Studies 1 and 2) addresses all four concerns.

The authors should at least explain how these concerns were addressed. Although the authors stated that “since the age did not differ between two ethnic groups, it is quite probable that the age would not moderate the observed tendencies” (p. 9), the authors did not have data on age. ^3^ It is unclear how the authors concluded that the age would not moderate the observed tendencies. Moreover, as stated above, the issue is not only about the group difference in the average age.

## 2. Can the name index be used as an individual-level indicator?

The authors treated the name indices as individual-level indicators reflecting personal inner characteristics. In presenting the results, they used the term “social cognition” throughout the text and “cognitive tendencies” (p. 10). Moreover, the authors suggested that Study 3 increased the “ecological validity” (p. 6) of the findings in Studies 1 and 2, which measured individual-level psychological tendencies.

However, the previous research the authors cited regarded name indices as group-level indicators that reflect group (e.g., nation, state, and culture) characteristics ( Ogihara et al., 2015; Twenge et al., 2010; Varnum & Kitayama, 2011). Because names can be determined by several individuals, such as couples, family members, and community members, naming involves a collective process of decision making. Thus, naming does not necessarily reflect individual psychology and behavior. For example, a husband may suggest a name, but his wife may reject it and choose a different name based on her mother’s advice. In this case, the husband’s psychology and behavior are not reflected in the name. Mongolians may ask lamas and/or elders to name their children. Thus, Studies 1 and 2 and Study 3 treated concepts at different levels. The authors should explain how they confirmed whether the name indices can be used as individual-level indicators. This level of concept (unit of analysis) is important when examining the relationship between culture and psychology (e.g., Cohen & Varnum, 2016; Schwartz, 2014; Vu et al., 2017).

## Conclusion

I have proposed two suggestions on Stojcic et al. (2020). I hope these suggestions will contribute to a better understanding of ethnic differences in names, psychology, and culture.

## Data availability

No data are associated with this article.

## Footnotes

^1 ^
It is necessary for the authors to explain why they conducted statistical hypothesis testing even though they had all data on the populations (all names of Chinese Mongolian and Han Chinese residents of the Inner Mongolia).

^2 ^
Similarly, there is another inconsistency between Studies 1 and 2 and Study 3: age group. In Studies 1 and 2, the authors collected data from university students. However, in Study 3, the authors collected data from all age groups who engaged in naming, and thus, their ages were unclear (not limited to the same age group as that in Studies 1 and 2). The pattern of ethnic difference might differ across age groups.

^3 ^
I asked the corresponding author whether the authors had data on the ages of name holders. The author replied that they did not.
