# Supplementary material for: Ethnic differences in names in China: A comparison between Chinese Mongolian and Han Chinese cultures in Inner Mongolia
Source: F1000Res. 2022 Jan 18;11:55. [Version 1] doi: 10.12688/f1000research.76837.1 (PMC9294497; doi:10.12688/f1000research.76837.1)
Supplement: Peer review history

# Peer review: Reviewer response for version 1

**DOI:** 10.5256/f1000research.80819.r143438

**Published:** 2022-07-18

**Authors:** Han-Wu-Shuang Bao

**Affiliations:** 1 Manchester China Institute, The University of Manchester, Manchester, UK

In the correspondence article, Ogihara raised two concerns about the validity of Stojcic et al.’s (2020) Study 3, which suggested that Chinese Mongolian (vs. Han Chinese) were more independent as they were less likely to give the top 1, 10, and 20 common names to babies. ^1^ I have conducted several studies on Han Chinese names, including one that confirms the increasing prevalence of unique names in China over 1920 to 2005 (Bao et al., 2021). ^2^ I also have carefully read the original article by Stojcic et al. (2020), the correspondence by Ogihara, and the other two reviewers’ insightful comments on this correspondence. In general, I agree with the two points proposed by Ogihara as well as most of the other two reviewers’ opinions. In my review report here, I first provide my further consideration of Ogihara’s two points and then propose three additional concerns about the original article. I hope my additional thoughts, together with Ogihara’s two important suggestions, would contribute to an even better understanding of both the cultural differences and the cultural changes of personal names, either within China or between countries.

**Further Consideration of Ogihara’s Two Points**

 Stojcic et al.’s (2020) Study 3 used an invaluable name database of the whole population of Chinese Han and Mongolian in the Inner Mongolia Autonomous Region of China. They computed the percentage of the most common first names given to each ethnic group separately by gender. However, as Ogihara pointed out, they did not control for the effect of birth cohort. Evidence has shown that Chinese names are becoming more unique over birth cohorts from 1920 to 2005 (Bao et al., 2021). If they could access a dataset with birth year information for each individual (e.g., the *2005 China’s 1% Population Census*, which I have used in my previous study; Bao et al., 2021), then this issue would be well addressed. Nonetheless, please also see the next section for my additional concerns, which suggest that even though the time effect was addressed, such a direct comparison between Han Chinese names and Chinese Mongolian names might be problematic.

 Ogihara’s second point concerns the level of analysis. I fully agree that the percentage of common names (hereinafter referred to as “PCN” for simplicity) is a group-level (rather than individual-level) index that has usually been utilized to study macro-level cultural changes (e.g., Twenge et al., 2010). ^3^ However, my own reading of the original article shows that they actually regarded PCN as “a national or cultural indicator of individualistic/collectivistic tendencies” (p. 6; Stojcic et al., 2020), which is appropriate. Thus, I don’t think this point of criticism is much necessary and to the point, though conclusions should of course be drawn with caution.

 Instead of limiting our discussion to these two concerns, I would like to share my three additional concerns about the original article. I hope both Ogihara’s and my concerns would facilitate the understanding of both cross-cultural differences and cross-temporal changes of names.

**Three Additional Concerns About the Original Article**

***(1) The percentage of common names (PCN) is not a proper index for Han Chinese names***

 First, before we discuss “Can the name index be used as an individual-level indicator?”, we have to ask “Can the name index be used as a group-level indicator?” In particular, is PCN a generally appropriate index to measure group-level name uniqueness? The answer is no. Twenge et al. (2010) validly measured English name uniqueness by the percentage of uncommon first names; Ogihara et al. (2015) ^4^ validly measured Japanese name uniqueness by the percentage of uncommon pronunciations paired with a Chinese character; Bao et al. (2021) validly measured Han Chinese name uniqueness by the name-character uniqueness in naming practice (negative log-transformed character frequency). Since naming norms vary substantially between cultures and languages, we have to use distinct measures to capture the uniqueness of names for a specific culture or language.

 For instance, PCN might not be a valid index of Han Chinese name uniqueness, because Han Chinese are allowed to use any single Chinese character, any reasonable combination of two characters, or even a possible combination of three characters to name their babies (Bao et al., 2021). In this case, Han Chinese names are not simply “selected” from an established name list, but rather “created” by combining different Chinese characters. Therefore, name-character uniqueness of a character combination reflects people’s psychological pursuit of uniqueness and is more proper to capture Han Chinese name uniqueness (Bao et al., 2021; Cai et al., 2018). ^7^ In contrast, English first names have a finite option list for people to select from, thus PCN is appropriate to measure English name uniqueness (Twenge et al., 2010). Meanwhile, a Japanese name can be paired with different pronunciations, which is a special naming norm that exists neither in China nor in English-speaking countries. Hence, pronunciation uniqueness is a specifically valid index of Japanese name uniqueness (Ogihara et al., 2015).

 In short, name-uniqueness indices are distinct, and thus not comparable, between cultures and languages. The PCN index used in Stojcic et al.’s (2020) Study 3 is valid to measure English name uniqueness but not so for Han Chinese names. Indeed, my unpublished data indicate that the PCN of Han Chinese names (combinations) showed a “decreasing-increasing-decreasing” pattern between 1930 and 2008, and the PCN reached the highest in the 1980s. Together with the changing pattern of name length (Bao et al., 2021), this merely suggests that more Han Chinese in the 1980s were given single-character (vs. multi-character) names (e.g., “伟”, “强”, “静”, “娜”; see also Tables 5 and 6 in Stojcic et al., 2020). The use of single-character names increases the level of PCN but does not necessarily suggest a preference for common or uncommon Chinese characters. For Han Chinese names, PCN largely depends on the number of characters in a given name, and thus is not much indicative of name uniqueness in a psychological sense (e.g., a psychological need for uniqueness).

***(2) PCN cannot be used for cross-cultural comparisons***

 Second, we move our focus to the comparison between Han Chinese and Chinese Mongolian names. The most common Mongolian names listed in the Tables 5 and 6 in Stojcic et al. (2020) were paired with *pinyin* (i.e., Han Chinese pronunciation). However, a Mongolian name written in Han Chinese characters is just a translated form based on its Mongolian pronunciation (i.e., transliteration). Thus, each Chinese character in a translated Mongolian name no longer has its own meaning, but rather should be considered with other characters to obtain the meaning in Mongolian. For example, the Mongolian name “乌兰图雅” actually consists of two indivisible words “乌兰” (which means “red” in Mongolian) and “图雅” (which means “sunglow” in Mongolian). In contrast, the corresponding Han Chinese name with the same meaning is “红霞”, which consists of two indivisible Chinese characters “红” (red) and “霞” (sunglow). Such linguistic differences constitute a critical factor that makes the PCN less comparable between these two ethnic groups.

 Furthermore, PCN is not comparable between cultures in general. For example, Twenge et al. (2010) showed that the PCN of the top 10 American boy names in 1950 was about 33% (see their Figure 1 at p. 21), whereas Su et al. (2016) found that the PCN of the top 10 Han Chinese boy names in 1950 was 18% (see their Table 3 at p. 35). ^5^ Could we therefore conclude that Han Chinese were more independent than American in 1950? Moreover, for the span of about 1950~2007, Twenge et al. (2010) showed the American boys top 10 PCN = 8~33% and the American girls top 10 PCN = 8~23% (see their Figure 1 at p. 21); in contrast, for “the span of some 60 years” (presumably 1950~2008), Stojcic et al. (2020) found the Han Chinese boys top 10 PCN = 4.18% (see their Table 5) and the Han Chinese girls top 10 PCN = 4.88% (see their Table 6). Obviously, Han Chinese were in general much less given “common names” than American. Is it thus reasonable to conclude that Han Chinese were more independent than American over the second half of the 21st century?

 In sum, it might be problematic to use PCN as an indicator of name uniqueness regardless of culture and then use it for cross-cultural comparisons. PCN can hardly capture the uniqueness of Han Chinese names, because Han Chinese names are much more complex than English names and could be more validly measured by using the name-character uniqueness (Bao et al., 2021).

***(3) Pursuing uniqueness is conceptually different from being independent***

 Finally, I would like to differentiate between the concepts of uniqueness, independence, and individualism. According to Oyserman et al. (2002), ^6^ individualism and collectivism are both multi-faceted constructs; “independent” and “unique” are two *parallel* domains of individualism. That is, being unique is different from being independent, though both indicate being individualistic. While “independence” highlights relying on oneself and being self-sufficient, “uniqueness” emphasizes seeking a distinctive identity and being different from others. Therefore, unique-naming practices are only indicative of the “uniqueness” domain/facet of individualism, but not indicative of the “independence” domain/facet. My unpublished data further showed that the cultural-level increase in name-character uniqueness in China can be specifically explained by the cultural-level emphasis on “uniqueness” (rather than “independence”). To this end, the group-level index of name uniqueness may be only regarded as an indicator of the uniqueness facet of individualism, instead of an over-generalized indicator of the other facets of individualism (e.g., independence, competition, autonomy, etc.).

 Taken together, the above three concerns, along with Ogihara’s two suggestions, may help us understand some nuances (1) between uniqueness and independence, (2) between different name-uniqueness indices for different cultures/languages, (3) between group-level and individual-level indicators, and (4) between Han Chinese and Chinese Mongolian names. I hope all these critical but constructive thoughts will advance the cultural psychological research using names as an index.

Are arguments sufficiently supported by evidence from the published literature or by new data and results?

Partly

Is the conclusion balanced and justified on the basis of the presented arguments?

Yes

Is the rationale for commenting on the previous publication clearly described?

Yes

Are any opinions stated well-argued, clear and cogent?

Partly

Reviewer Expertise:

Cultural psychology, Cultural change, Individualism, Name, China

I confirm that I have read this submission and believe that I have an appropriate level of expertise to confirm that it is of an acceptable scientific standard.

## References

[1] : Historical Sustenance Style and Social Orientations in China: Chinese Mongolians Are More Independent Than Han Chinese. *Front Psychol* .2020;11: 10.3389/fpsyg.2020.00864 864 10.3389/fpsyg.2020.00864 32457683PMC7225263

[2] : Novel Evidence for the Increasing Prevalence of Unique Names in China: A Reply to Ogihara. *Front Psychol* .2021;12: 10.3389/fpsyg.2021.731244 731244 10.3389/fpsyg.2021.731244 34938229PMC8685573

[3] : Fitting In or Standing Out: Trends in American Parents' Choices for Children’s Names, 1880–2007. *Social Psychological and Personality Science* .2010;1(1) : 10.1177/1948550609349515 19-25 10.1177/1948550609349515

[4] : Are common names becoming less common? The rise in uniqueness and individualism in Japan. *Front Psychol* .2015;6: 10.3389/fpsyg.2015.01490 1490 10.3389/fpsyg.2015.01490 26557100PMC4613833

[5] : The Evolution of Given Names and Times Change (in Chinese). *Youth Studiesl* .2016;3:31-38

[6] : Rethinking individualism and collectivism: Evaluation of theoretical assumptions and meta-analyses. *Psychological Bulletin* .2002;128(1) : 10.1037/0033-2909.128.1.3 3-72 10.1037/0033-2909.128.1.3 11843547

[7] : Increasing Need for Uniqueness in Contemporary China: Empirical Evidence. *Front Psychol* .2018;9: 10.3389/fpsyg.2018.00554 554 10.3389/fpsyg.2018.00554 29867619PMC5952033

# Peer review: Reviewer response for version 1

**DOI:** 10.5256/f1000research.80819.r120384

**Published:** 2022-03-09

**Authors:** Kenta Tsukatsune

**Affiliations:** 1 Tokyo Metropolitan University, Tokyo, Japan

*General Evaluation*

 Overall, the reviewer can mostly agree with arguments of this paper. Author Ogihara's discussion about the analysis in Study 3 of Stojcic et al.'s (2020) paper on how to handle cohort/age groups, and relationships of the individual and group indicators are both acceptable. ^1^ The reviewer also has read the original paper, and as the author points out, particularly analytical confusion and ambiguity in definitions of generation and time series are noticeable in Study 3. Names from a wide range of different age groups are lumped together and compared, when careful handling of birth years is fundamentally important in name data, and the chronological relationship between phenomenon of individualization and appearance of names (the birth year of a person) is treated carelessly. The reviewer does not think it is necessary to change the author's points themselves, and is fine with the relatively favourable wording for the original paper ("Addressing these two questions would contribute to a better understanding...").

 However, there are some important issues as well as the two aforementioned issues that are not pointed out in this paper. The following contents are mainly not inadequacies in this paper, but the original paper, but these matters can be included in this paper to enhance its validity. If there are any of the following items that the author agrees with, the reviewer would like to have them included partly, even if they are not all of them.

 Study 3 of the original paper leaves many questions unanswered in terms of cultural differences in language and naming. In particular, there are issues related to differences in ideographic and phonetic scripts, and the relationship between names of ethnic minorities including Inner Mongolia and culture of Han Chinese. This is not to argue that it is inappropriate to make comparisons, but rather that we should carefully examine the basis for whether comparisons can be made. Without much consideration of the assumptions, the original paper talks about comparing names as an extension of the analysis of psychological questionnaire (generally, variables obtained from questionnaire are much more comparable than variable of names).

*About letters used to write languages*

 The author of this paper has himself done careful research on the differences between ideographic and phonetic naming systems, and the reviewer hopes that the author will include this point (and that it is at least an unavoidable issue in discussion about the control of age groups).

 First, an important feature of Han Chinese naming is that in addition to the vocabulary that the name itself indicates as a single lump of words, the component Chinese characters often have unique meanings (while English names are less clearly aware of the elements that make up the word), and Chinese characters themselves are ideographic. On the other hand, Inner Mongolian naming is considered to have inherited culture of Mongolian language and nomenclature. It can be said that Chinese Mongolians apply Chinese characters to names derived from their phonetic character culture. In this regard, a full explanation from a linguistic point of view should be given as to whether the changes in names of Mongolians can be compared with those of Han Chinese, which have been formed by ideographic character culture (even if we take into account the fact that Mongolians in China also partly adopt the same naming methods as Han Chinese). If we compare this to studies of names in the English-speaking world, when a comparison is made between European-Americans and African-Americans, the two groups are compared on basis of a common linguistic foundation of naming according to English, even though they have various different cultural backgrounds. In contrast, names of the two groups mentioned in the original paper (Tables 5 and 6) seem to be two groups with distinctly different language systems, and it is difficult to consider them without a linguistic explanation. Also, in terms of the number of characters (number of Chinese characters), Mongolian names are longer, and the reviewer wonders if those changes can be treated in the same way as the changes in Han Chinese names, which usually consist of one or two characters.

*About relationships between ethnic groups and their naming systems*

 Next, the reviewer would also like to point out that the relationship between ethnic minorities and ethnic majorities should be carefully examined. Naming system themselves, as well as housing policies and economic means, are prone to political and legal changes, and influenced by relationships between ethnic groups. Between groups that follow the same system, comparisons of individualization could be made, comparing names from different generations or different ethnicities. However, in the case of Inner Mongolians and Han Chinese, it is not clear whether the underlying systems themselves are similar enough to be lined up immediately, or whether, if there is heterogeneity, the analysis in the original paper has been able to control for it.

 To the best of the reviewer's knowledge, original naming systems of the two cultures are considered to be very different. Han Chinese naming system consists of a general first name and a family name, while Mongolian culture uses the father's name in a position like a family name, alongside first name. And, in the case of Inner Mongolians, complications exist in the process of deciding what to adopt as the first and last name on resident registration using Chinese characters.  It seems that some people have two names, one in Han Chinese style and the other in Mongolian style, and then register one of them officially. There are also cases where only a part of the Mongolian-derived name is used as a family name. There are variations in the explanations in different literature and articles, but in any case, whether Mongolian baby names are equivalent to Han Chinese baby names after registration must be thoroughly discussed. For example, even if names are becoming more personalized, there may be not only differences in the degree of personalization itself, but also problems that do not exist in the Han Chinese, such as how to use Chinese characters for pronunciation and which part of the name should be used as the family name.

 The following literature briefly explains the linguistic differences between Mongolian and Chinese names.

 Yunshaab, S. (2019), “Survey of Natural Language Processing for Vertical Mongolian: Current Situation,” Status of NLP for Vertical Mongolian, Report 1, viewed 1 March 2022( ^2^, DOI: 10.5281/zenodo.4536516)

 The following literature explains dual name practices. It also shows that there are differences in the use of Han Chinese style names depending on friendships, generation, gender, social class, etc. ^3^

 The following web page provides explanations of personal names in Inner Mongolia by a number of experts and people who have lived there ( Link, Viewed 1 March 2022).

*About chronological changes in naming*

 The above discussion on writing systems and lexicon, and the relationships of ethnic groups is not unrelated to the author's point about cohort/age group. For example, in Han Chinese, a particular Chinese character is often used in a particular cohort, but is there a corresponding phenomenon in names of Inner Mongolians? If such a phenomenon does not exist, or if it does exist but it is linguistically completely different from the case of Han Chinese, which are based on ideographic characters, then we must first discuss the comparability of the two. And, as for the names of Inner Mongolians, is it not possible that Han Chinese style names are increasing as generations progress?

*About research methods for variables and indicators by levels*

 It seems to the reviewer that the current state of this paper is sufficient for pointing out about the indicators of individual-level and group-level. However, if there is room in the paper, can the author suggest some improvements? What about suggestion of calculating correlations between variables at individual level, or between variables at group level? For example, several studies in Europe and the United States have conducted analyses that link characteristics of individual names with the psychological and behavioural characteristics of the individuals. It would be possible to keep only characteristics of the name (for example, whether the name is in the top ranking or not) as a variable and analyse the relationship between it and psychological characteristics of the person. On the other hand, if names are to be used only as group indicators, it would be possible to create more detailed group indicators for each region, and calculate the correlation of the indicators for individualism and settlement in each region with the trend of names in that region.

 Finally, let me make it a condition for this paper to pass that the author must include one or more remarks, in some form or another, about the lack of linguistic explanation (can we really compare Han and Mongolian names?) in the analysis of the original paper. I think this is something that must be included as a point against the handling of the data set, and this is a topic that is closely related to the topic of age groups. Also, I would like to see the ethnic relations included, but it is not a requirement.

 It is not necessary to include all of my suggestions, and the author can use his own ideas on the matter. However, a handling of these issues would substantially improve the quality of the paper.

Are arguments sufficiently supported by evidence from the published literature or by new data and results?

Yes

Is the conclusion balanced and justified on the basis of the presented arguments?

Partly

Is the rationale for commenting on the previous publication clearly described?

Yes

Are any opinions stated well-argued, clear and cogent?

Partly

Reviewer Expertise:

Onomastics, social statistics

I confirm that I have read this submission and believe that I have an appropriate level of expertise to confirm that it is of an acceptable scientific standard, however I have significant reservations, as outlined above.

## References

[1] : Historical Sustenance Style and Social Orientations in China: Chinese Mongolians Are More Independent Than Han Chinese. *Front Psychol* .2020;11: 10.3389/fpsyg.2020.00864 864 10.3389/fpsyg.2020.00864 32457683PMC7225263

[2] : Status of NLP for Vertical Mongolian. *Zenodo* .2021; 10.5281/zenodo.5103499 10.5281/zenodo.5103499

[3] : Urban Mongols: Ethnicity in Communist China. *preprint* . Reference source

# Peer review: Reviewer response for version 1

**DOI:** 10.5256/f1000research.80819.r120385

**Published:** 2022-02-14

**Authors:** Ivana Stojcic, Xiaopeng Ren

**Affiliations:** 1 Key Laboratory of Behavioral Science, Institute of Psychology, Chinese Academy of Sciences, Beijing, China; 2 The Laboratory of Social and Engineering Psychology, Institute of Psychology, Chinese Academy of Sciences, Beijing, China

In the Ethnic differences in names in China: A comparison between Chinese Mongolian and Han Chinese cultures in Inner Mongolia—Commentary, the author poses two main questions i.e. the issue of time control in baby names analyses and the issue of the unit level analysis, and a minor question related to inferential statistics. The main central concern of the time control in baby naming practices is subdivided into following questions: control of confounding factors, the true meaning of indicators, past vs. present sustenance styles, and use of different study approaches, i.e. the analysis of data over a period of 60 years vs. 1 year.

**1. How was the effect of time controlled?**

 When discussing the issue of the lack of time control in baby name analyses, it is important to note that it was initially recognized and pointed out in the Limitations section of the published manuscript Historical sustenance style and social orientations in China: Chinese Mongolians are more Independent than Han Chinese, albeit in less detail compared to the arguments raised in this Commentary. The following was stated considering this issue: “… *while we followed the recommendation of Twenge’s and Varnum’s method (Twenge et al., 2010; Varnum and Kitayama, 2011), there’s one difference between our approach and theirs. We used the most common names for the population not at 1 year. Nonetheless, since the age did not differ between two ethnic groups, it is quite probable that the age would not moderate the observed tendencies. Future studies should address this issue to further verify our findings and provide more detailed insight into reported tendencies.” ^1^*

 Ogihara makes a good point in arguing that analyses performed in Study 3 did not exclude the possibility for observed differences in name giving practices to derive from other confounding factors rather than ethnic differences. Unlike previous studies, which analyzed the respective changes in name practices by decade or per year, ^2^ ^3^ we analyzed the baby naming practices for a period of some 60 years, thus blending other potential confounding factors such as age and generation. While our approach fails to provide insight into changes in the naming practices throughout decades, it still gives valuable contribution to the research question we wanted to explore.

 Furthermore, the author questions the meaning of the presented indicators, i.e. top 1, 10 and 20 most common names calculated against the period of 60 years, by arguing that common names can drastically change over time and that common names 60 years ago may no longer be considered as common in the present, and the meaning of such indicator may be somewhat ambiguous. While we agree with the author’s statement that names can change over time, and that more precise data would be obtained with analyzing naming practices in one given year, as previously stated, this change in name practices is not usually as drastic as the author suggests. In fact, previous studies have shown that preferences that become quickly popular also tend to lose that same popularity very fast, and this trend has also been observed in naming practices where names that quickly gained popularity, died faster and were less likely to persist. ^4^ Further support for this premise comes from baby naming practices in Croatia, where according to available statistical data (see here), the 1st most common male and female names, which were respectively Ivan and Marija, remained unchanged for 4 decades, i.e. since 1929 – 1959, after which they dropped to 3rd and 5th place, respectively, with male name Ivan re-gaining the 1st place in 1980 – 1999. Consequently, it is reasonable to assume that an indicator composed of 60 years of name giving practices still reveals valuable data on behavioral tendencies and orientation toward individualism/collectivism.

 Again, related to the use of data roughly covering a period of 60 years, Ogihara suggests that we contradicted ourselves by arguing that past sustenance styles affect “present” independence/interdependence (individualism/collectivism) but then proceed with examining past psychological tendencies from more than 60 years ago. We assume that the author misunderstood what we tried to say, as previous studies have shown that cultural differences based on historical subsistence style continue into the modern era, even in areas where most people no longer engage in the traditional subsistence activity but work in modern industry. ^5^ By using data that comprise a period of 60 years, we provide full support for what we argue for, as these data do not cover specific time point 60 years ago but are comprehensive of six decades to the present day, thus reflecting recent and current behaviors. Furthermore, even if for example we solely focused on year 1960, our reasoning would still be correct as historical subsistence style refers to subsistence patterns dating back far beyond the past century. ^6^

 Finally, we fully support the Ogihara’s suggestion that it would be more precise to analyze only name indicators for recent years as was done in previous studies that used name giving practices as indicators of individualism, ^7^ ^2^ which here was not possible due to lack of available data stratified by year. Nonetheless, when interpreting reported finings, it is also important to consider that examined ethnic groups were in similar age groups and generations, which may somewhat decrease the possibility of confounding during this period. Furthermore, both of these ethnic groups underwent very similar social and political changes, which also ensured certain kind of control over a variety of confounding factors, but naturally not all. In a different study that used the percentage of most common names for the period 1981-2010 to explore the spatial-temporal changes in individualism, the uniqueness among the provinces followed the similar changing pattern along the year, which was also consistent with the results reported for prefectures in Japan and States in US. ^8^ These data suggest that the time would not have such a strong effect on provincial differences to reverse the observed direction and differences between groups.

 Future studies should investigate the name giving practices within the same year, as this is the most precise and correct approach for calculating the individualism indicator. We are also open to the idea of changing and revising our conclusion and rejecting our hypothesis if new refined evidence, inconsistent with our findings, should emerge. However, the present results that were based on available data seem to suggest that the current hypothesis is the most possible explanation to the subject matter under consideration.

**2. Can the name index be used as an individual-level indicator?**

 When discussing the second main concern raised by Ogihara, it is necessary to clarify few things. First, the phrase “increase the ecological validity” was meant to say that as the results from Study 3 were reflection of real life behavior, they had greater ecological validity compared to the results reported in Study 1 and Study 2. We assume that Ogihara focuses on ecological validity, as he was expecting behavioral tendencies toward individualism/ collectivism to be measured by an indicator in real setting similar to symbolic self-inflation used in Study 1 and 2. In view of this assumption, the Study 3 is not perfectly designed, but is definitely relevant to the overall research. The chosen methodology, i.e. the percentage of most popular name, is undoubtedly an indicator of uniqueness, which is one the facets of individualism. ^9^ While uniqueness is not perfect equivalent to self-inflation, some items of the Singelis’ self-construal scale used to measure self-inflation in Study 1 and 2, focus on uniqueness, e.g. “I enjoy being unique and different from others in many respects”. We admit that this is not optimum connection; nonetheless, it is relevant one.

 In addition, Ogihara argues that naming practices do not reflect individual psychology or behavior at individual level as they may result from a collective process that includes babies’ parents and extended kinship. We agree with Ogihara’s view to a certain extent, considering that baby naming practices are usually the results of the collective decision between minimally two people. Nonetheless, each one of these individuals participating in such a decision is also motivated to give or suggest a common or unique name him/herself. As stated by Ruth Fulton Benedict "culture is personality writ large". ^10^ While naming practices provide valuable source of information that mirror parents’ values or orientations, thus making them suitable behavioral measure encompassing important choices and decisions of parents, ^7^ they are also a valuable cultural product, which can successfully reveal societal orientation tendencies, as well as cultural change. ^2^ ^3^ ^7^ In fact, even though group differences occur because individuals are motivated to strive for uniqueness, thus lowering the percentage of common names ^3^ as groups are made up of individuals, and as there are reciprocal influence processes between individuals and bigger groups, it is impossible to consider one, without the other. ^11^

 We also assume that the author was concerned with the potential issue of ecological fallacy. ^12^ ^11^ In general, there’s risk of ecological fallacy when differences observed at individual level are simply translated to explain differences at group level, and vice versa. For example, when one takes a relationship that has been established between two or more variables at one level of analysis and then assumes that this proves something at a different level of analysis. ^11^ However, considering the baby name practices, it can be assumed or implied that the same motivation underlies the uniqueness at individual level and group level. If each individual in one cultural group is generally motivated to be unique or stand out from the whole group, this would increase the number of uncommon baby names at individual level, which would eventually result in the low percentage of the common names. Likewise, if each individual in other cultural group is generally motivated to be similar with others and to fit in the group, he/she would be inclined to give the baby a common name at individual level, which would eventually result in high percentage of most common names. In fact, few scholars have also developed alternative indicators of uniqueness related to baby naming practices following the same logic. E.g., in Japan, Chinese characters are used to name babies; however, they can be diversely pronounced. Ogihara constructed the indicator of uniqueness based on uncommon pronunciation of a Chinese character, and used it to explore the changes in uniqueness trends in Japan (Ogihara). ^7^ ^13^ Cai also constructed the indicator of uniqueness based on the frequency of Chinese characters in names and used it to explore the changes in uniqueness trends in China. ^14^ He also constructed a uniqueness of nickname in Sina weibo based on the use of non-Chinese characters to explore the provincial differences in individualism within China. ^15^ Ogihara furthermore used dog naming practices to evaluate whether Japanese culture became more individualistic. ^16^  As our main intent was to explore the Mongolian/Han differences in individualism/ collectivism through different indicators, our research was a combination of three different studies, and more importantly all of our assumptions were based and backed by relevant results.

 Finally, with reference to the issue of the use of inferential statistics test, we agree with Ogihara that inferential statistics test is not necessarily required when population is used instead of sample, and as we used the entire population of Inner Mongolia autonomous zone in the present study, it was not necessary to use inferential statistics. In fact, the first draft of the manuscript did not include the inferential statistic, which was subsequently included following the suggestion of one of the reviewers. Nonetheless, as inferential statistics generated consistent results, they were not further discussed.

**Conclusion**

 To sum up, we fully agree with Ogihara’s suggestion that future studies should investigate the name giving practices within the same year so as to further validate reported findings. As previously stated, we are also open to the idea of changing and revising our conclusion and rejecting our hypothesis if new refined evidence, inconsistent with our findings, should emerge. However, the present results that were based on available data seem to suggest that the current hypothesis is the most possible explanation to the subject matter under consideration.

Are arguments sufficiently supported by evidence from the published literature or by new data and results?

Partly

Is the conclusion balanced and justified on the basis of the presented arguments?

Partly

Is the rationale for commenting on the previous publication clearly described?

Yes

Are any opinions stated well-argued, clear and cogent?

Partly

Reviewer Expertise:

Cultural and social psychology; differences in individualism and collectivism

We confirm that we have read this submission and believe that we have an appropriate level of expertise to confirm that it is of an acceptable scientific standard.

## References

[1] : Historical Sustenance Style and Social Orientations in China: Chinese Mongolians Are More Independent Than Han Chinese. *Front Psychol* .2020;11: 10.3389/fpsyg.2020.00864 864 10.3389/fpsyg.2020.00864 32457683PMC7225263

[2] : Fitting In or Standing Out: Trends in American Parents' Choices for Children’s Names, 1880–2007. *Social Psychological and Personality Science* .2010;1(1) : 10.1177/1948550609349515 19-25 10.1177/1948550609349515

[3] : What's in a name?: popular names are less common on frontiers. *Psychol Sci* .2011;22(2) : 10.1177/0956797610395396 176-83 10.1177/0956797610395396 21196534

[4] : How adoption speed affects the abandonment of cultural tastes. *Proc Natl Acad Sci U S A* .2009;106(20) : 10.1073/pnas.0812647106 8146-50 10.1073/pnas.0812647106 19416813PMC2688844

[5] : On the Origins of Gender Roles: Women and the Plough *. *The Quarterly Journal of Economics* .2013;128(2) : 10.1093/qje/qjt005 469-530 10.1093/qje/qjt005

[6] : Large-scale psychological differences within China explained by rice versus wheat agriculture. *Science* .2014;344(6184) : 10.1126/science.1246850 603-8 10.1126/science.1246850 24812395

[7] : Are common names becoming less common? The rise in uniqueness and individualism in Japan. *Front Psychol* .2015;6: 10.3389/fpsyg.2015.01490 1490 10.3389/fpsyg.2015.01490 26557100PMC4613833

[8] : Social economic development and spatial-temporial shifts of individualism within China 1981-2010.

[9] : Rethinking individualism and collectivism: Evaluation of theoretical assumptions and meta-analyses. *Psychological Bulletin* .2002;128(1) : 10.1037/0033-2909.128.1.3 3-72 10.1037/0033-2909.128.1.3 11843547

[10] : Ruth Fulton Benedict (1887-1948). *Women anthropologists: A biographical dictionary, Gacs, U; Khan A. McIntyre, J. and Weinberg, R. (eds.)* .1988;1-7.

[11] : Levels of Analysis in Cross-Cultural Psychology. *Online Readings in Psychology and Culture* .2002;2(2) : 10.9707/2307-0919.1018 10.9707/2307-0919.1018

[12] : Culture's Consequences:International Differences in Work-Related Values. *Sage Publications* .1980;5:

[13] : Temporal Changes in Individualism and Their Ramification in Japan: Rising Individualism and Conflicts with Persisting Collectivism. *Front Psychol* .2017;8: 10.3389/fpsyg.2017.00695 695 10.3389/fpsyg.2017.00695 28588512PMC5440576

[14] : Increasing Need for Uniqueness in Contemporary China: Empirical Evidence. *Front Psychol* .2018;9: 10.3389/fpsyg.2018.00554 554 10.3389/fpsyg.2018.00554 29867619PMC5952033

[15] : Climato-Economic Origins of Variations in Uniqueness of Nickname on Sina Weibo. *Front Psychol* .2021;12: 10.3389/fpsyg.2021.599750 599750 10.3389/fpsyg.2021.599750 34054633PMC8155360

[16] : Are people giving more unique names to their dogs? Rising uniqueness and individualism in Japan. *Paper Session Presented at the 62nd Annual Meeting of Japanese Group Dynamics Association (Nara)* .2015;
